# Anastomosis of Free Flap Pedicle to Great Vessels

**DOI:** 10.29252/wjps.7.3.351

**Published:** 2018-09

**Authors:** Jalaluddin Khoshnevis, Terifeh Dashti, Mohammad Ebrahimi, Eznollah Azargashb, Mohamadreza Kalantar Motamedi

**Affiliations:** 1 General and Vascular Surgery, Shohada-Tajrish Medical Center, Shahid Beheshti University of Medical Sciences, Tehran, Iran; 2 Health Service Management, Clinical Research Development Center of Shohada-Tajrish Hospital, Shahid Beheshti University of Medical Sciences, Tehran, Iran; 3 Department of Community Medicine, Shahid Beheshti University of Medical Sciences and Health Services, Shohada-Tajrish Hospital, Tehran, Iran

**Keywords:** Free jejunal, Flap, Great vessels, Latissimus

## Abstract

**BACKGROUND:**

Free Flaps are viable option to cover the tissue defect. Pedicle anastomosis to vessel branches has excellent result. In some situations which there is a possibility of flap failure like shortage of vessel branches, possibility of pedicle kinking or need to vein graft, anastomosis to great vessels is justified.

**METHODS:**

Six patients were allocated to study. Five cases for free jejunal flap and one case for free latissimus flap. In free jejunal flap group, pedicle anastomosis was performed as an end-side fashion to common carotid artery and internal jugular vein and in free latissimus flap, pedicle was anastomosed as an end-side fashion to superficial femoral artery and superficial femoral vein. Follow up was regular up to 20 years.

**RESULTS:**

In free jejunal flap group, there were three female and two male with age from 30 to 59 years. The sixth case was a thirteen years old male with flexion contracture of right knee who underwent free latissimus flap. Follow up was regular for 20 years. All flaps survived, and good functional result was obtained in all except one.

**CONCLUSION:**

Choosing great vessels as one side of anastomosis is safe and can be done as a primary approach due to technical demand or as a final resort when there is shortage of side branches.

## INTRODUCTION

Free jejunal flap was the first free flap described in the literature. From Seidenberg report at 1959 to date, remarkable advances have been achieved in the technique with overall success of 95-97%.^1^^-^^3^ The tube lengths of up to 30 cm has been harvested and replaced lost esophagus successfully.^4^ The second jejunal branch is usually the best site for flap harvesting providing arterial pedicles up to 3-4 mm in diameter. All arterial and venous anastomosis were done to side branches of subclavian or carotid artery and jugular veins.^1^^-^^4^

Few anastomosis were done to external carotid artery.^5^ Vein grafts were accused as one of the causes of flap failure.^6^ Also failures was reported from venous kinking.^6^ Choosing the recipient vessels is still a challenge.^7^^-^^10^ Many factors influence the choice of recipient vessels. History of head and neck radiation, surgery, and type of reconstruction dictates the choice of recipient vessels. It goes without saying tributaries are the first choice avoiding the possible complication by choosing the great vessels as recipient vessels. Here, we wish to introduce safety of the anastomosis to great vessels as recipient vessels to expand the choices and avoid additional dissections in new or complicated cases.

## MATERIAL AND METHODS

Six patients were allocated to free flap with anastomosis of its pedicle to great vessels. Five out of six underwent free jejunal flap. After harvesting of the flap, side-side anastomosis was undertaken between jejunum and pharyngoesophagus. Then, we attended the position of the pedicles, which at the most of them were lying on the common carotid artery and internal jugular vein. Totally, 100 IU/KG of heparin was injected as IV bolus before harvesting of flap. 

The pedicle opening was spatulated three times of their diameter and anastomosed as an end-side fashion to common carotid artery and internal jugular vein with prolene 8/0 under 3x loupe magnification. Little maneuver such as pedicle dissection at the mesentry or orientation of arteriotomy and venotomy may be required for good adjustment. Proximal opening of the jejunal tube was spatulated and anastomosed to the side of pharyngotomy or closed primarily. The distal ostomy was closed two month later after complete healing of the wound.

## Results

We had excellent results in four of five free jejunal flaps and complete relief of dysphagia. In one patient, the failure was due to severe scar tissue of pharyngeal region due to burn injury and breakdown of pharyngojejunal anastomosis and consequently the wound. Despite this problem, the flap survived for two months. The six^th^ case who underwent free latissimus flap for knee contracture had good results with normal walking (Table 1).

**Table 1:** Demoghraphic characteristics of the patients.

**Case**	**Sex**	**Age (year)**	**Pathology**	**Type of flap**	**Vessel anastomosis to**	**Follow up (year)**	**Complications**	**Figure**
1	F	59	Anastomosis stricture after orringer operation.	Free jejunal flap	Common carotid artery internal jugular vein	5	No	
2	F	55	Unresectable esophageal web through endoscopy	Free jejunal flap	Common carotid artery internal jugular vein	10	No	2 and 3
3	F	50	Stricture due to epidermolysis bullosa	Free jejunal flap	Common carotid artery internal jugular Vein	20	No	4 and 5
4	M	47	Stricture due to epidermolysis bullosa	Free jejunal Flap	Common carotid artery internal jugular vein	8	No	6 and 7
5	M	30	Stricture due to caustic agent	Free jejunal flap	Common carotid artery internal jugular vein	2 Months	Wound dehiscence	-
6	M	13	Knee contracture after burn injury	Free latissimus flap	Superficial femoral artery superficial femoral vein	10	No	8-10

**Fig. 1:**
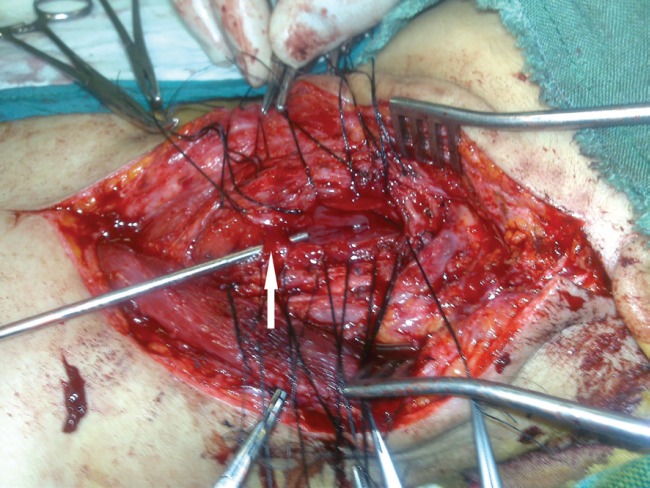
Sever stricture (arrow).

**Fig. 2:**
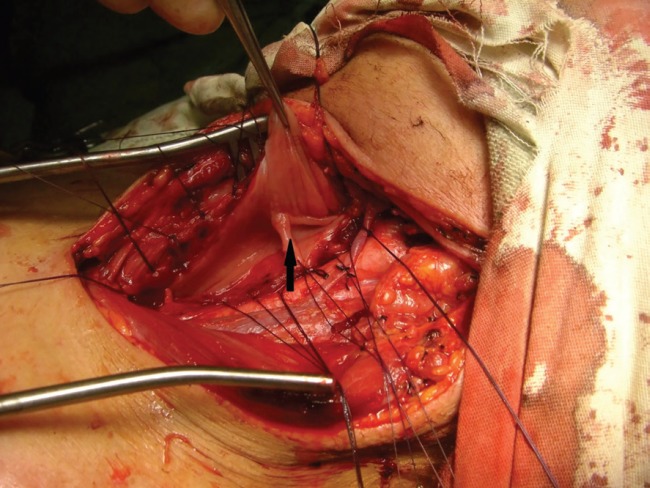
Esophageal web (arrow).

**Fig. 3:**
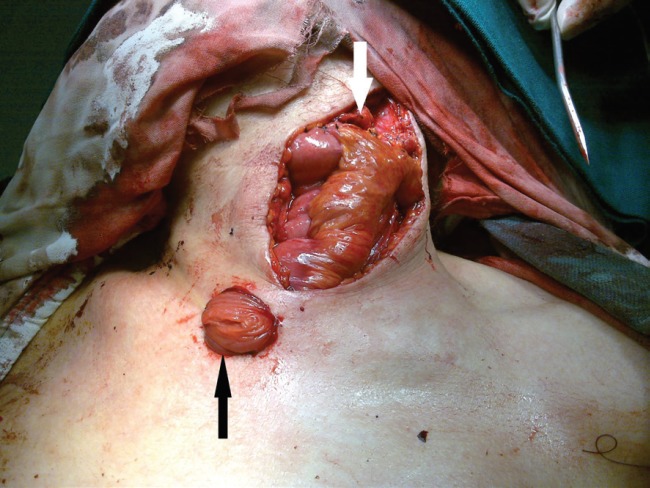
Proximal end (white arrow) ,distal end (black arrow).

**Fig. 4:**
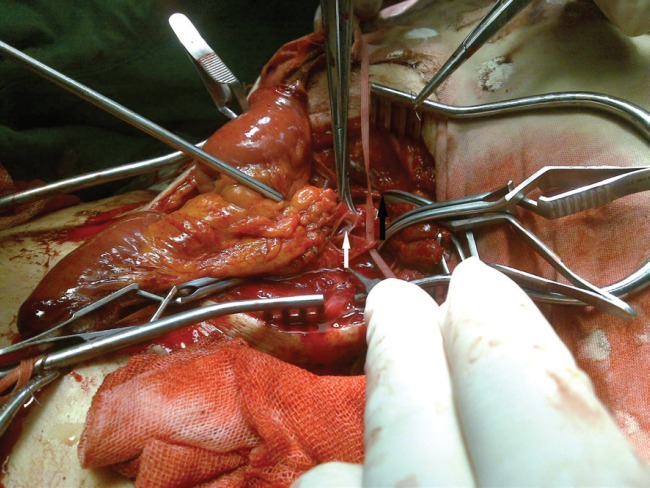
End-side pedicle vein to internal jugular vein anastomosis (white arrow) and end-side pedicle artery to common carotid artery anastomosis (black arrow)

**Fig. 5:**
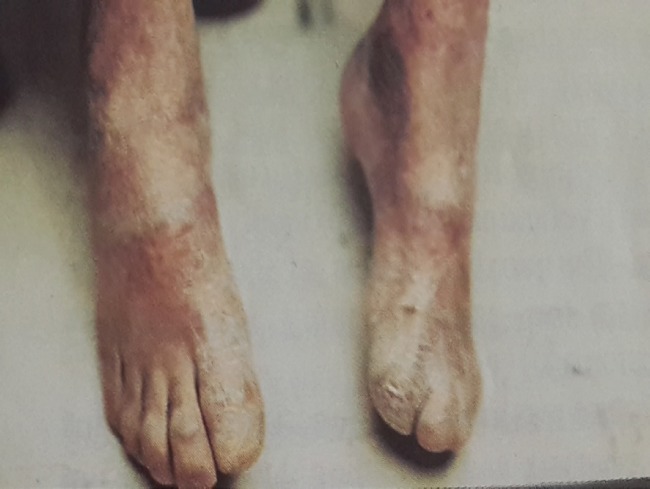
Nail destruction and web

**Fig. 6:**
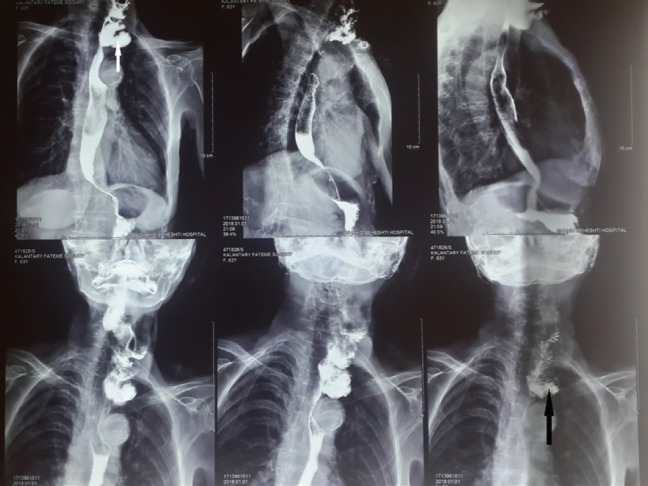
Blind loop during barium swallow (white arrow) and during evacuation by reverse peristaltism (black arrow) after 8 years

**Fig. 7:**
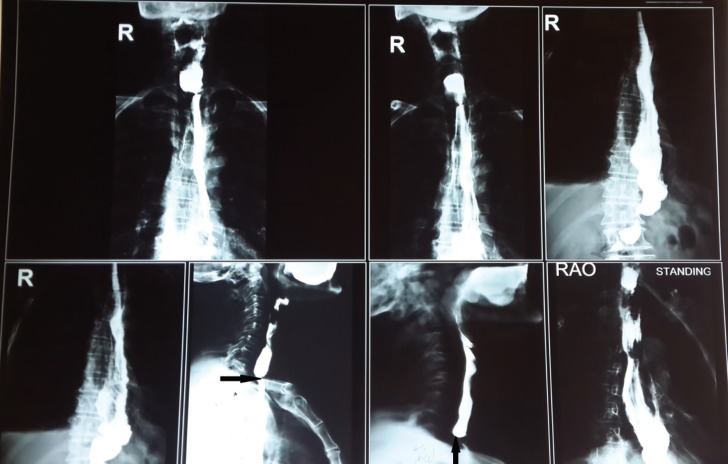
Esophageal stricture (arrows)

**Fig. 8:**
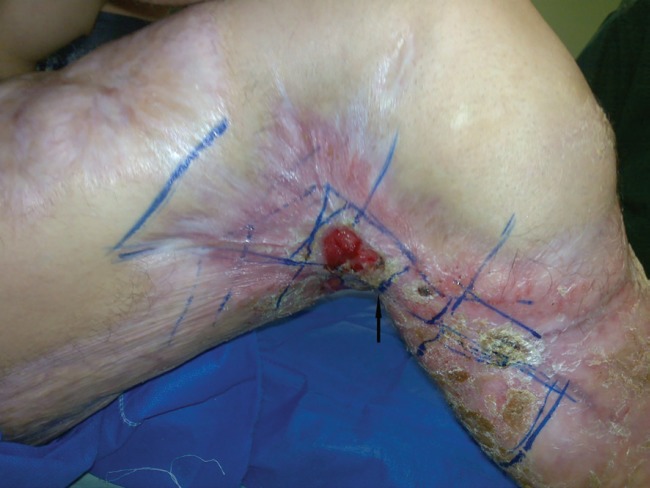
Flexion contracture of knee (arrow)

**Fig. 9:**
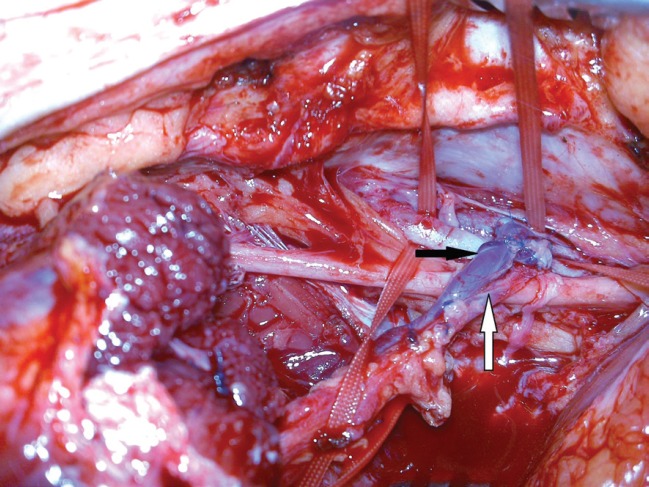
Anastomosis to SFA (white arrow) and SFV (black arrow)

**Fig. 10:**
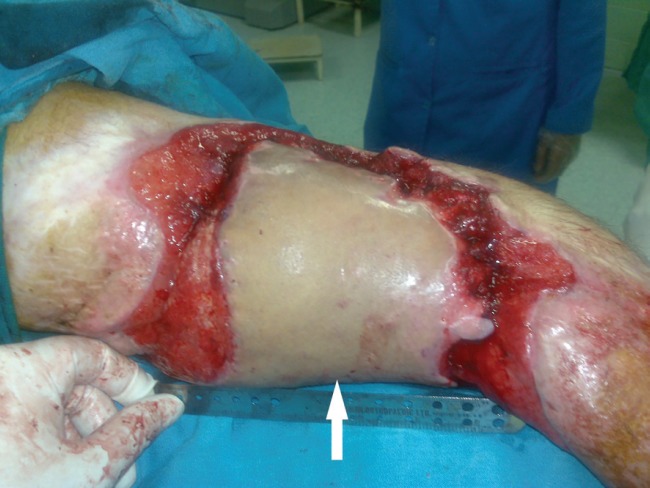
Latissimus dorsi flap after vein anastomosis revision (arrow)

## DISCUSSION

Free jejunal flaps were used as tubular flap to substitute pharyngoesophageal defect or were opened to replace the mouth floor defect. In our cases, the jejunum was used as a tube, which was opened by its side and anastomosed to the side esophagotomy. After side-side esophagojejunal anastomosis, the pedicle lie was evaluated just the moment, which laid in the middle of common carotid artery and internal jugular vein. Many authors ignored this ideal situation for anastomosis due to potential complication of anastomosis to common carotid artery and makes tributaries as first recipient vessels.^10^^-^^14^

This is a true logic but when the tributaries have been destroyed due to previous surgery and radiation, you may go far for seeking new recipient vessel. This needs more times and dissections and many need vein graft to accomplish the procedure, which ultimately may lead to more failure.^15^^-^^18^ Our approach to common carotid artery as first recipient showed to be a safe one and the results was comparable to other authors.^19^^-^^21^ We had no flap failure, but only a wound dehiscence. The flap survived for two months before the next plan. Side-side esophagojejunal anastomosis proved to be a best one, because we had no dysphagia and stenosis during their long follow-up. 

Considering two cases of epidermolysis bullosa cases who were prone to stenosis adds to the merit of this type of anastomosis. End to distal side esophagotomy prohibited due to more dysphagia occurrence.^21^ In the six^th^ case who had the history of sever burn injury and four surgeries to treat the knee contracture, we decided to treat with free latissimus flap. There were paucity of recipient tributary and thick femoral vein wall due to inflammation. We had no choice except end-to-side anastomosis to femoral artery and vein. 

After flap congestion, the wound was explored and a small thrombosis was removed behind the femoral vein anastomosis. Drainage problem relieved by punching the vein and re-anastomosis. Despite this problem, the flap survived and knee contracture was relieved. After ten years, walking was normal. At this site, also the vessel recipient of choice was the tributaries.^22^^,^^23^ Few studies were found to mention anastomosis to the major vessels as recipient.^2^ All of them had good results.^24^^-26^ We look forward for further cases to find a new place for this type of anastomosis at this site in algorithm.

In our study, anastomosis of pedicle of free flaps to great vessels was justified. Though anastomosis to branches has its own merit, there is situation like paucity of branches due to burn, surgery, radiotherapy or need for a vein graft or possibility of kinking in which the incidence of flap failure rises. So direct anastomosis of the pedicle to the side of common carotid artery and internal jugular vein was shown to be safe and worthy.
